# Using an adaptive, codesign approach to strengthen clinic-level immunisation services in Khayelitsha, Western Cape Province, South Africa

**DOI:** 10.1136/bmjgh-2020-004004

**Published:** 2021-03-24

**Authors:** Andrea Timothy, David Coetzee, Christopher Morgan, Margaret Kelaher, Ross Stewart Bailie, Margie Danchin

**Affiliations:** 1 Research School of Population Health, Australian National University, Canberra, Australian Capital Territory, Australia; 2 Melbourne School of Population and Global Health, The University of Melbourne, Melbourne, Victoria, Australia; 3 Faculty of Health Sciences, University of Cape Town, Observatory, Western Cape, South Africa; 4 JHPIEGO, Baltimore, Maryland, USA; 5 University Centre for Rural Health, The University of Sydney, Lismore, New South Wales, Australia; 6 Infection and Immunity, Murdoch Children's Research Institute, Parkville, Victoria, Australia; 7 Department of Paediatrics, The University of Melbourne, Melbourne, Victoria, Australia; 8 Department of General Medicine, The Royal Children's Hospital, Melbourne, Victoria, Australia

**Keywords:** child health, health education and promotion, health services research, health systems, immunisation

## Abstract

**Introduction:**

Optimal immunisation programme service delivery and childhood vaccine coverage remains an ongoing challenge in South Africa. Previous health systems approaches have made recommendations on how to address identified barriers but detailed local implementation studies are lacking. This study aimed to improve immunisation service delivery in children under 24 months in Khayelitsha, Western Cape Province using an adaptive, co-design approach to assess and improve childhood immunisation service delivery at the clinic level.

**Methods:**

A rapid, adaptive approach to identification of barriers and assessment of current childhood immunisation service delivery was developed with three clinics in Khayelitsha, Western Cape Province. This informed a short co-design process with key stakeholders and service providers to develop local interventions targeted at high priority barriers. Interventions were implemented for 4–6 months and evaluated using theory-based evaluation tools. Clinic service delivery, satisfaction and changes to clinic processes and parent engagement and knowledge were measured.

**Results:**

Interventions developed included weekly community immunisation education radio sessions, daily clinic health talks, immunisation education and promotion materials and service provider and parent quality checklists. Evaluation post-intervention showed improvement in parents’/guardians’ knowledge about immunisation, parent engagement and service provider commitment to improvement in service quality. Radio sessions and immunisation education and communication materials were deemed most useful by parents and providers.

**Conclusion:**

Immunisation service delivery can be strengthened using an adaptive, clinic-led assessment process which can effectively identify barriers, inform co-designed interventions and be evaluated over a short period. This approach provides a framework to guide future local participatory action research to more effectively improve childhood immunisation service delivery and other child health services in under-resourced settings.

**Key questions**

**What is already known?**

- Optimal immunisation service delivery remains an ongoing challenge in South Africa with stagnating immunisation coverage for children under 1 year of age. Responses are needed to address these barriers to service delivery at all levels, including at the local clinic level.
- A rapid, co-design approach to immunisation service delivery, intervention development and implementation targeted to identified barriers at the clinic level has not been reported previously in South Africa.
- The most similar approach is the WHO tailoring immunisation programmes (TIP) approach which focuses on identification of barriers and potential strategies to improve childhood immunisation uptake; however, this is usually led by government or national authorities, and a clear locally useful process for implementation or evaluation of strategies was often lacking.

**What are the new findings?**

- Co-designed, local-level interventions resulted in improvement in parents’/guardians’ knowledge about immunisation, parent engagement and service provider commitment to improvement in immunisation service quality.
- Providers and clients had clear preferences for quality improvement tools, prioritising radio sessions and take-home communication materials

**Key questions**

**What do the new findings imply?**

- Rapid experienced-based co-design approaches to generate rapid service delivery improvements at the clinic level may prove feasible elsewhere in South Africa and other low-income and middle-income countries (LMIC) settings.
- The approach could be enhanced with a ‘project champion’ and/or an ‘implementation team’ to ensure ongoing delivery, assessment and evaluation of routine immunisation services and expansion of the approach to include other primary healthcare clinic services, such as HIV/TB care, to optimise Universal Healthcare (UHC).
- Development of a simplified ‘toolkit’ and scaled down approach based on the process used in this study could assist clinics or implementation teams and represent a valuable addition to the WHO TIP approach, for immunisation at the clinic level in LMIC settings.

## Introduction

The South African Expanded Programme on Immunisation (EPI) has had considerable impact on vaccine-preventable diseases, contributing to polio eradication, tetanus elimination and improved control of measles, pertussis and diphtheria. However, as in many low-income and middle-income countries (LMICs), optimal immunisation service delivery remains an ongoing challenge.^1 2^ This is particularly true in the large partially informal townships in the Western Cape Province such as Khayelitsha, Cape Town where there are significant access issues and frequent migration.^3^ Immunisation coverage for all EPI vaccines for children under 1 year of age in the City of Cape Town is estimated to be 87%, well short of national targets.^4^

South Africa’s challenges and barriers to high immunisation coverage are similar to other LMIC settings, representing an important constraint to accessing universal healthcare (UHC).^5^ Despite recommendations for improving the immunisation programme and health system more broadly, there is a limited evidence base in South Africa on how recommendations have been implemented and demonstrated to improve service delivery.^2 3 6–8^ Initial WHO Tailoring Immunisation Programmes (TIP) approaches have focused on a comprehensive and often lengthy process of identifying barriers and potential strategies,^8^ however, more timely and potentially effective strategies at the local clinic level have not been implemented or evaluated in South Africa.

Two emerging research disciplines were considered relevant to potentially address this research gap and inform a new approach to developing and implementing interventions to improve service delivery: Implementation Research and Delivery Science (IRDS) and experience-based co-design (EBCD).^9^ IRDS allows for the translation of theoretical knowledge into practical, context-specific and evidenced-based approaches and EBCD gathers information on service users’ experiences to identify barriers and facilitators to programme delivery. EBCD also includes collaborative ‘co-design’ with both providers and users designing solutions, facilitated by a third party.^10 11^

There has been limited use of IRDS and EBCD to improve immunisation service delivery in South Africa or internationally.^12 13^ This study, called the ‘Khusela Immunisation Study’, aimed to use aspects of these approaches to develop a rapid, adaptive approach to strengthen immunisation service delivery at the clinic level for children under 24 months in Khayelitsha.

Phase 1 of the study, which included an initial assessment of barriers and facilitators to immunisation service delivery using surveys, focus groups, key informant interviews and observation. This assessment, incorporated service provider and service user perspectives and an audit of clinic data and processes to identify barriers to service delivery. The major barriers to service delivery that were identified mainly related to issues with vaccine data quality, concerns about access or practical barriers to immunisation, parent engagement and knowledge, and quality of service. Phase 1 will be published separately.

This paper describes the second and third phases of the study which include the co-design of local interventions, implementation and evaluation.

## Methods

### Study design

This phase of the Khusela Immunisation Study was a cross-sectional, pre-post mixed-methods study completed in the subdistrict of Khayelitsha, Western Cape Province, South Africa between June 2017 and May 2018. A whole-of-systems approach consisting of two components was used:

1. Co-design of local-level interventions to improve immunisation service delivery, with key stakeholders. This component included prioritisation of barriers to be addressed, and a feasibility review of strategies to implement a suite of interventions.
2. Evaluation, incorporating service provider and service user perspectives, and an audit of clinic data and processes to identify changes made to service delivery. A process evaluation component was included in this phase which assessed changes made to interventions during the implementation period compared with what was initially planned, adjustments made to interventions and why, as well as parents’/guardians’ identification of what they liked or disliked about the interventions, and what additional information they thought was needed.

The approach used in this study combined elements from various systems strengthening approaches, policy and evaluation frameworks and assessment tools, most heavily drawing from IRDS and EBCD.^9–21^ These approaches were adapted and applied at the clinic level with the overall aim of providing a holistic solution to address barriers to immunisation service delivery which encompasses co-design and implementation of local, targeted interventions and the evaluation of their effectiveness. A mixed-methods approach for data collection and analysis was used, combining both quantitative and qualitative methods to enhance validity and enrich findings.^22^ This is summarised in online supplemental figure 1 and online supplemental table 1.

10.1136/bmjgh-2020-004004.supp1 Supplementary data

10.1136/bmjgh-2020-004004.supp2 Supplementary data

This manuscript follows SQUIRE V.2.0 standards for reporting.^23^

### Setting and participants

Three public health clinics providing child health services in Khayelitsha were approached and agreed to participate in the study (clinic A, clinic B and clinic C). These clinics provide standard primary healthcare services including immunisation, HIV and TB care with the bulk of care delivered by registered nurses and enrolled nurses. The clinics ranged in size, with clinic A seeing approximately 1000–1500 primary healthcare patients under the age of 5 years per month, clinic B approximately 2000–2500 per month and clinic C approximately 600–1000 patients per month.

Key stakeholders in the initial stages of the co-design process included representatives from WHO South Africa and the Western Cape Department of Health, who supported the project. The project working group consisted of two representatives from the City of Cape Town Health and service providers from the three clinics (three facility managers, six nurses, six clerks). Three focus groups, conducted during the final stages of the intervention development process, consisted of 20 service users (parents/guardians of children aged under 24 months old who attended the clinics).

For the subsequent evaluation of interventions post-implementation, service providers were included if they were directly involved in either management or administration of EPI service delivery and interacted directly with parents/guardians. Radio station staff were included if they were involved in the delivery of the weekly radio sessions that had been implemented. Service users were included if they were parents/guardians of children aged under 24 months old who attended the clinics. Purposive sampling of EPI service users was used to include parents/guardians attending the clinics based on their willingness and availability to participate in the survey while in the clinics’ waiting area. A subset of these participants were randomly selected to participate in focus groups.

Prior to the commencement of focus groups and interviews, parents/guardians and service providers were given plain language statements that included a brief description of the study and study outcomes. Written informed consent was obtained from all participants.

### Co-design: intervention development and implementation

Findings from the assessment stage of the study identified a range of key barriers that could be addressed through tailored interventions (reported in a separate publication).

**Figure 1:**
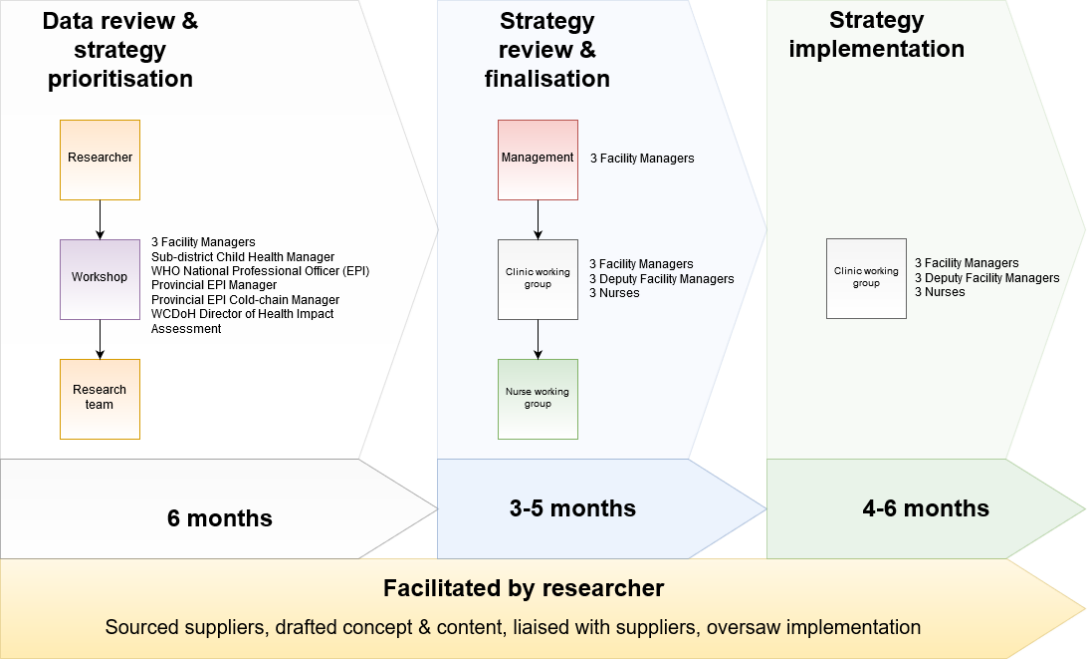
Strategy development and implementation process.

There were two stages in the intervention co-design process (figure 1):

1. Data review and prioritisation of identified barriers (duration: 6 months): was conducted post phase 1 of the study and discussed in a 1-day workshop consisting of key stakeholders (provincial and subdistrict management, facility management and clinic staff) and the research team. Workshop outcomes included three identified interventions prioritised by the key stakeholders based on feasibility, acceptability and necessity for development by each clinic.
2. Intervention review and finalisation (duration: 3–5 months): was carried out by an initial working group consisting of the three clinics’ facility managers and the primary researcher was convened to finalise selection of the interventions, including addition of a fourth intervention. Subsequent weekly working groups included nurses from each clinic and the primary researcher to assist with the development process for each intervention. Final intervention refinement occurred via focus groups with service users at each clinic.

### Evaluation: post-implementation

#### Data collection

##### Pre–post survey

Structured surveys were conducted in isiXhosa with parents/guardians of children aged under 24 months old at the clinics. The assessment tools used were adapted from key sources to suit the South African context, mainly tools to assess vaccine hesitancy and equitable healthcare.^14 18 24^ Questions related to parent/guardian perceptions, and concerns about vaccines and experiences with immunisation service delivery. The initial assessment survey was conducted approximately 6 months prior to the co-design process.

A second survey was conducted four to 6 months post-implementation focusing on changes parents/guardians had seen in immunisation services, as well as any changes in knowledge, perceptions and concerns about vaccines.

##### Post-implementation interviews and focus group discussions

Four to six months after intervention implementation, semistructured interviews and focus groups were conducted in English with service providers and radio station staff, and in isiXhosa with parents/guardians. Assessment tools used were adapted from health systems approaches that were deemed appropriate for the local context.^14–18^ Questions further expanded on information gathered during the surveys, and delved into changes seen across the clinics and the impact of the intervention implemented.

#### Outcomes

The outcomes measures across all data collection methods included:

1. Clinic service delivery outcomes, including the number of EPI vaccine doses delivered to children under 9 months per month (diphtheria-tetanus-pertussis-3, measles-containing-vaccine-1 (MCV1), pneumococcal conjugate vaccine 3 and MCV2).
2. Parent engagement and knowledge, including changes in parent/guardian attitudes towards immunisation and interaction between parents/guardians and service providers.
3. Parent satisfaction with immunisation services provided.
4. Changes to clinic processes, including accessibility of services.
5. Effectiveness of implementation of the interventions, as assessed by parents and service providers

#### Data management and analysis

Survey data were collected and managed using REDCap (Research Electronic Data Capture) hosted at the Murdoch Children’s Research Institute, Melbourne, Australia. Interviewers used the REDCap Mobile App for offline data collection and uploaded to the online database thereafter. Survey data were cleaned and collated before being exported to SPSS v25 for analysis. The difference in survey responses pre-intervention and post-intervention implementation were calculated using proportion tests, 95% CIs and p values (significant difference if <0.05). χ^2^ tests were used to test any associations between intervention components and any differences.

Key themes and changes in perspectives post-intervention implementation were identified from interviews and focus groups with service providers and parents/guardians. Subthemes identified from core themes, and any patterns emerging from coded data were analysed using NVivo to assist with data management.

### Patient and public involvement

Patient and members of the public (referred to in this paper as service providers and service users) were involved in various stages of this study. Following phase 1 of the study, data were disseminated to provincial and subdistrict management, facility management and clinic staff in order to prioritise barriers to service delivery that needed to be addressed. All interventions were then co-designed with clinic staff, which included facility management, nurses and clerks, and were then further refined in collaboration with a group of parents/guardians of children aged under 24 months old who attended the clinics.

## Results

### Interventions developed through the co-design process

Four interventions were prioritised and developed through the co-design process. These interventions were in place at each of the clinics for a period of 4–6 months (see online supplemental figures 2–4).

10.1136/bmjgh-2020-004004.supp3 Supplementary data

10.1136/bmjgh-2020-004004.supp4 Supplementary data

10.1136/bmjgh-2020-004004.supp5 Supplementary data

#### Weekly community radio sessions

Weekly 1-hour radio sessions focused on all aspects of childhood immunisation and were presented by a qualified nurse and immunisation nurse from one of the study clinics, who rotated weekly. The nurses presented for the first 30 min, followed by listener questions for the second half an hour.

#### Nurse-led education sessions at each clinic

Daily 15 min immunisation talks were conducted in clinic waiting rooms. Talks were led during the morning session by a qualified nurse or immunisation nurse as parents arrived before clinic started, and were continued by community care workers throughout the day. Sessions included a question-and-answer component. There were approximately four sessions per day.

#### Service provider and parent quality checklists

Service provider checklists were developed for nurses and clerks as a prompt to optimise delivery of all components of the immunisation sessions and were linked to the parent checklists. English and isiXhosa parent checklists were provided to gather feedback on immunisation service quality and user experience, and used as an education tool for parents to improve awareness of expectations from immunisation sessions. Checklists were based on the Myanmar Collaborative Community Checklists for Immunisation^25^ and the WHO Immunisation Session Checklist^26^ and facilitated by community care workers in clinic waiting areas.

#### Health promotion materials

Four A2 posters were designed by the research team with a local graphical designer based on identified knowledge gaps for parents which addressed the booking process and lengthy waiting time, the EPI vaccine schedule and vaccine preventable diseases, and vaccine safety concerns post immunisation (potential adverse events following immunisation). Four matching English and isiXhosa A6 postcards were provided for parents to take home.

### Post-implementation evaluation

#### Characteristics of evaluation participants

Interview and focus group participants comprised 47 EPI service providers, which included 2 representatives from the City of Cape Town Health, 3 health facility/deputy health facility managers, 6 immunisation nurses, 3 pharmacists, 6 clerks and 26 community care workers.

A total of 369 service users (parents/guardians) participated in surveys (n=352) and 4 focus groups (n=17) and 3 radio staff in a focus group. The sociodemographic characteristics of survey participants from the clinics are detailed in table 1. The demographic make-up of the cohort recruited was similar to that of the subdistrict of Khayelitsha as detailed in data from the most recent South African National Census of 2011, apart from the sex ratio which in our sample was 95% female and 3% male as compared with almost 50–50 female–male in the Census data.^27^

**Table 1:** Sociodemographic characteristics of survey participants (n=352)

Variable	N (%)
Gender	
Female	**335 (95)**
Male	11 (3)
Age (years)	
Range=14-64	
Mean 27.68 (SD 6.85)	
Median=27	
<20	15 (4)
**20–24**	**79 (22)**
**25–29**	**94 (27)**
**30–34**	**81 (23)**
35–39	55 (16)
40–44	17 (5)
45–49	5 (1)
≥50	1 (0.3)
Marital status	
Father of the child does not live with you, but supports you or the child	28 (8)
Father of the child lives with you	28 (8)
Married	104 (30)
Mother of the child does not live with you, but supports you or the child	2 (0.6)
Mother of the child lives with you	2 (0.6)
Single father	5 (1)
**Single mother**	**177 (50)**
Widow	3 (1)
Province/country of birth	
Eastern Cape	**192 (55)**
Free State	1 (0.3)
Gauteng	14 (4)
KwaZulu-Natal	6 (2)
Northern Cape	2 (0.6)
Western Cape	120 (34)
Zimbabwe	15 (4)
Language(s) spoken at home	
English	2 (1)
**isiXhosa**	**341 (97)**
isiXhosa and isiZulu	1 (0.3)
isiXhosa and Sotho	3 (1)
isiZulu	3 (1)
Shona	13 (4)
Sotho	5 (2)
Highest level of education	
Never attended school	3 (1)
Preschool	2 (1)
Completed primary school	10 (3)
**Completed secondary school**	**178 (51)**
Some secondary school	107 (30)
University/further education	46 (19)
Type of dwelling	
Backyard dwelling	34 (10)
**Brick house**	155 (44)
**Shack**	158 (45)
Other	2 (0.6)
No of children	
**1**	**155 (44)**
2	102 (29)
3	66 (19)
4	20 (6)
≥5	5 (1.6)
Age of youngest child range=2 months-12 years	
0–6 months	53 (15)
**7–12 months**	**151 (43)**
13–18 months	81 (23)
19–24 months	47 (13)
>24 months	17 (5)

#### Clinic service delivery outcomes

Overall, there was no significant change in the number of vaccine doses administered per month before and after intervention implementation with minor % differences per vaccine administered. However, an increase was noted in the total number of vaccines administered per month (4% at clinic A, 12% at clinic B, 6% at clinic C), and in MCV2 at all clinics ranging from a 6%–12% increase (see online supplemental table 2).

#### Parent engagement and knowledge outcomes

There was an increase in parents/guardians agreeing they had sufficient knowledge to make decisions about immunising their child following intervention implementation (pre 57% vs post 75%; difference 18%; p=0.118). While not statistically significant, the trend suggested that the interventions may have had a positive impact on parents’/guardians’ knowledge about immunisation or the immunisation programme.

Parents’/guardians’ views remained predominantly supportive of the importance of the EPI programme (pre 98% vs post 99%), and almost all participants agreed that vaccines were important for their child as in the preintervention assessment (pre 98% vs post 99%).

Significant improvement was noted in parent/guardians reporting, they felt comfortable with how they were treated at the clinics (pre 50% vs post 78%; difference 29%; p=0.01). There was also a significant decrease in parents/guardians who felt uncomfortable (pre 44% vs post 13%; % difference= −31%; p≤0.0001) (see table 2).

**Table 2:** Parental/guardian report of interaction with service providers, accessibility and satisfaction with services

	Pre-intervention (n=427) n (%)	Post-intervention (n=352) n (%)	Total (n=779) n (%)	Difference % (95% CI)	P value (χ^2^ test)
Parental/guardian report of interaction with service providers					
Parents comfortable with the way they are treated at the clinic					
Yes	213 (50)	276 (78)	489 (63)	29 (27 to 30)	0.01
No	185 (44)	46 (13)	231 (30)	−31 (−32 to to 28)	<0.0001
Unsure	25 (6)	27 (8)	52 (7)	2 (2 to 2)	0.576
Reasons parents are comfortable with the way they are treated at the clinic					
Friendly staff	154 (36)	173 (49)	327 (42)	13 (12 to 14)	0.16
Helpful staff	174 (41)	196 (56)	370 (47)	15 (14 to 16)	0.13
Clinic is welcoming	136 (32)	127 (36)	263 (34)	4 (4 to 4)	0.61
No concerns about confidentiality	153 (36)	70 (20)	223 (29)	−16 (−17 to to 15)	0.03
Clear explanation received from staff	137 (32)	118 (34)	255 (33)	1 (1 to 2)	0.86
Other	4 (1)	6 (2)	10 (1)	1 (1 to 1)	0.64
Reasons parents are not comfortable with the way they are treated at the clinic					
Unfriendly staff	112 (26)	19 (5)	131 (17)	−21 (−22 to to 20)	<0.0001
Unhelpful staff	101 (24)	16 (5)	117 (15)	−19 (−20 to 18)	0.0003
Clinic is not welcoming	116 (27)	13 (4)	129 (17)	−23 (−25 to to 22)	<0.0001
Concerns about confidentiality	46 (11)	11 (3)	57 (7)	−8 (−8 to to 7)	0.04
No clear explanation received from staff	89 (21)	17 (5)	106 (14)	−16 (−17 to to 15)	0.002
Other	9 (2)	11 (3)	20 (3)	1 (1 to 1)	0.66
Clinic service delivery: accessibility and parent/guardian satisfaction					
Communication from clinics to remind parents of appointments or follow-up when appointments are missed					
Yes	37 (9)	45 (13)	82 (11)	4 (4 to 4)	0.38
No	385 (91)	293 (83)	678 (87)	−30 (−30 to to 30)	0.54
Unsure	1 (0.2)	10 (3)	11 (1)	3 (2 to 3)	0.09
Feel that waiting times have been reduced					
Agree	N/A	205 (58)	N/A	N/A	N/A
Disagree		115 (33)			
Unsure		22 (6)			
Service delivered appropriately and effectively					
Yes	186 (44)	200 (57)	386 (50)	13 (13 to 14)	0.19
No	183 (43)	109 (31)	292 (37)	−12 (−12 to to 11)	0.16
Unsure	55 (13)	37 (11)	92 (12)	−2 (−2 to to 2)	0.67
Satisfaction with immunisation services provided at the clinic					
Very satisfied	216 (51)	314 (89)	530 (68)	39 (37 to 41)	0.001
Unsatisfied	174 (41)	17 (5)	191 (25)	−36 (−38 to to 34)	<0.0001
Unsure	34 (8)	15 (4)	49 (6)	−4 (−4 to to 4)	0.25
Able to let the clinic know if they are not satisfied with the service provided					
Yes	308 (72)	93 (26)	401 (51)	−46 (−48 to to 43)	<0.0001
No	84 (20)	208 (59)	292 (37)	39 (37 to 41)	<0.0001
Unsure	34 (8)	46 (13)	80 (10)	5 (5 to 5)	0.28
Parent preferred methods of providing feedback					
Complaints box	254 (60)	65 (19)	319 (41)	−42 (−44 to to 39)	<0.0001
Parent quality checklist	N/A	2 (0.6)	N/A	N/A	N/A
Talk to nurse	119 (28)	54 (15)	107 (14)	−13 (−13 to to 12)	0.05
Talk to doctor	53 (12)	28 (8)	202 (26)	−4 (−4 to to 4)	0.37
Talk to facility manager	174 (41)	18 (5)	54 (7)	−36 (−38 to to 34)	<0.0001
Talk to receptionist	36 (8)	21 (6)	72 (9)	−2 (−2 to to 2)	0.59
Talk to community care workers	51 (12)	23 (7)	74 (9)	−5 (−6 to to 5)	0.24

N/A, not available.

#### Changes to clinic processes

Over half of the parents/guardians surveyed said they felt waiting times at the clinics were reduced (58%). The largest difference seen was in overall parents’/guardians’ satisfaction (pre 51% vs post 89%; % difference=39%; p=0.001) and parents/guardians that were unsatisfied (pre 41% vs post 5%; % difference = −36%; p≤0.0001) (see table 2).

Overall, clinic procedures remained mostly the same pre-intervention and post-intervention implementation, and appointment systems were still in place. However, following the nurse working groups, all clinics implemented fast-tracked immunisations for children due for their 9-month immunisation (process summarised in online supplemental figure 5).

10.1136/bmjgh-2020-004004.supp6 Supplementary data

Qualitative data indicated that many parents/guardians identified changes that seemed to make the clinic more accessible:

‘I have noticed the changes in appointment[s]. It makes things easier… you don’t queue when you come for your appointment’ (Clinic A, parent/guardian 4).

One community care worker said that clinic staff seemed more conscious of following up on clients with an increase in the number of parents recalled by community care workers and in follow-up reminder phone calls.

There was also an expansion of the community care workers’ role in clinic B beyond home visits already in place. To improve client follow-up, they were given access to the Patient Record and Health Management Information System (PREHMIS):

I think sometime last year … we were introduced to the system, so those [children] who do not have [immunisations, that is, have missed appointments] we call the mother. We just go in the system [PREHMIS] then we click there, show the numbers [vaccine dose administered] and then everything appears, … we [see] if the child is fully immunised or not (Clinic B, CCW 1).

In the pre-intervention assessment, community care workers mentioned they felt underused. However, at clinic B, it appeared that dialogue improved post-intervention implementation between clinic staff and community care workers, and more responsibility was given to community care workers.

#### Feasibility, acceptability and reach of different interventions

For service providers, overall assessment was very positive for all interventions. Three out of four interventions were well received by parents/guardians who felt that the clinics should continue to support them. Overall, just under half of parents/guardians reported they listened to the radio sessions presented by the clinics (42%), heard the health talks in the clinics (43%) and had seen the health promotional materials (44%). The only intervention that had limited reach to parents/guardians were the quality checklists with most parents/guardians reporting they had not seen them (see table 3).

##### Weekly radio sessions

The majority of parents/guardians and service providers gave positive feedback and were aware of the radio sessions. Many service providers commented that when conducting home visits, parents/guardians indicated they heard about immunisation on the radio. One service provider appreciated the feedback from parents/guardians who called in, as a guide to quality improvement priorities. They felt that the clinics should continue to present the radio sessions as a means of direct education of listeners and also as a means for peer-to-peer information sharing. Staff at clinic B were highly enthusiastic and created a participatory environment where both clinic staff and parents/guardians attending the clinic were involved every Tuesday, either by reminding each other that the session was on, playing the radio over loudspeaker in the clinic, or clients listening to the radio using mobile phone in speaker mode so others in the waiting areas could listen in. The radio station staff were very positive about the radio session presented by the nurses. They shared that it would be a good idea to expand the radio sessions to other clinics from different areas, as while they were based in Khayelitsha, their listeners were from all over Cape Town (see table 4).

**Table 3:** Parents’ opinions about interventions that were implemented

	N (%)
Heard about immunisation on the radio	
Yes	146 (42)
No	89 (25)
Unsure	110 (31)
Thought radio sessions were useful	
Yes	142 (97)
Unsure	1 (0.6)
Thought clinics should continue presenting radio sessions	
Yes	142 (97)
Unsure	1 (0.6)
Heard health talks about immunisation in clinics	
Yes	152 (43)
No	142 (40)
Unsure	49 (14)
Thought health talks were useful	
Yes	149 (98)
No	1 (0)
Unsure	2 (1)
Thought clinics should continue presenting health talks	
Yes	149 (98)
No	1 (0)
Unsure	1 (0)
Seen posters/pamphlets about immunisation in clinics	
Yes	154 (44)
No	124 (35)
Unsure	64 (18)
Thought posters/pamphlets were useful	
Yes	149 (97)
No	1 (0.6)
Unsure	1 (0.6)
Thought clinics should continue using posters/pamphlets	
Yes	145 (94)
No	5 (3)
Unsure	1 (0.6)
Seen immunisation feedback form in clinics	
Yes	8 (2)
No	318 (90)
Unsure	22 (6)
Thought feedback forms were useful	
Yes	8 (100)
Thought clinics should continue using feedback forms	
Yes	7 (88)
No	1 (12.5)

**Table 4:** Outcomes of interventions based on parent/guardian and service provider comments

Intervention	Outcomes	Parent/guardian/service provider quote
Weekly radio sessions	Encouraged people to attend clinic	‘They are useful because there are people who don’t come to the clinic so it might encourage them to come to the clinic’ (Clinic C, parent/guardian 5).
	Educated regarding importance of immunisation	*‘…*some of [the parents] when I come [to the clinic]… they said… ‘I overhear from the radio that immunisation is very important that is why I came here’ (Clinic B, CCW 2).
	Allowed for feedback to be provided to clinics Encouraged service quality improvement	*‘…*they are so amazing because we used to get a good feedback of the kinds of things but we also used to get the bad feedback… it’s very important for us to get the bad feedback, that’s where we're going to try to raise our socks up’ (Clinic A, clerk 1).
	Built relationship between parents/guardians attending clinics and service providers	*…*it was a nice feeling to have the staff from this facility going to the radio… the clients feel so good because they know this person who speaks on the radio… on the Tuesday that she’s going to do the talks, [the nurse] was standing [in the waiting area] and telling [the parents] that ‘I’ll be doing the talk on the radio, you must listen’… others are coming with their earphones in case they are still here by that time, they would listen… we had one of our clients, she opened the radio and then she puts on the mic, and that way everybody would listen when she talks… (Clinic B, facility manager).
	Addressed gap in health promotion activities	*…*we have NGOs that are dealing with the other diseases like your HIV, TB, cancer and other ones, but immunisation it’s very rare to find organisations that are dealing with that. So, it was really helpful for our people, and we know that in black communities some parents may not take it seriously, that they have to take their kids for immunisation to the clinics… So, they need to know that information which is going to be helpful for their kids*…* (Radio staff 1).
	Introduced the possibility of expansion for other clinics from around Cape Town to be involved	*‘…*this idea of rotation from different clinics can help… not only Khayelitsha. Because the questions that [parents] used to ask… these nurse that they used to come here… were not able to tackle… because they are not working there’ (Radio staff 2).
Nurse-led health talks	Educated regarding importance of immunisation Allowed for linkage with other interventions that were implemented	‘[the nurse] was talking about the importance of immunisation and what you must do if you are bringing your child for immunisation. She even showed us a chart talking about the effects of not immunising your child’ (Clinic B, parent/guardian 5).
	Encouraged dialogue between parents/guardians and service providers Fostered an environment where parents/guardians acted as health promoters to the community	They ask the question, and then we answer those questions… they are used to talk to us to visit those people for those child who are not updated with their immunisation… Even the seniors… that hear the information… they go back to their homes with that information that are given at the clinic (Clinic B, CCW 1).
Service provider and parent quality checklists	Encouraged service quality improvement	*…*it is good because it is like a link between a clinic and the person so whoever is doing the survey will take it to the facility manager, and when there is this survey the clinic is going very quick…’ (Clinic C, CCW 1).
	Allowed for feedback to be provided to clinics	Initially, when they [the parents] were asked do you know… [and if] they didn’t know… I found that, ‘okay, this is the area where I should put emphasis on with the education’… those feedbacks… they help us to know where we should focus our talks…it was one of our tools to evaluate this [information] that we are giving out, does it sink well or need to put more… emphasis on that certain topic…so I think it a good thing to evaluate (Clinic A, facility manager).
	Not used to their full capacity during implementation period	‘They were not utilised effectively. I think that it’s not mandatory from the officers… people don’t take it seriously… Or if there is no one that is monitoring it, that is making sure you tick, please, you return the [checklist]’ (Clinic C, deputy facility manager).
Health promotion materials	Used as a supplementary information source to road to health card/booklet	‘I have seen this, I remember they were there by the security and each and every parent was taking from there, and to their road to health cards there are others that are stapled on their cards’ (Clinic C, CCW 4).
	Simplified educational information	Sometimes [parents] are lazy to read if it just a black and white. So, because there were nicely printed in colour and they’ve got the pictures as well, so they were well presented. Even if you don’t like to read, but the colour will draw you to just watch and read. And the information inside there was very good and it was simple and anyone can understand. (Clinic A, facility manager).

##### Nurse-led health talks

Some parents/guardians who heard immunisation health talks at the clinics described the sessions in detail and also mentioned that nurses or community care workers used materials to illustrate the talks. Service providers said that immunisation health talks were a large component of their role. Compared with parents’/guardians’ attitudes pre-intervention implementation; service providers felt that parents/guardians were now more engaged with health talks, and took more interest in ensuring that the people they knew had their child immunised as well (see table 4).

##### Quality checklists

Although the service provider and parent quality checklists were used for a short period of time at the clinics, most service providers felt they were useful and supported ongoing use.

###### Parent quality checklists

Many parents were unaware of the quality checklists as the clinics ran out of them and were unable to replenish supply, meaning that they were only distributed for 3 months. Given evaluation timing, it was likely that the cohort of parents/guardians who participated in focus groups did not have adequate exposure to the parent checklists.

###### Service provider quality checklists

Service providers who were receptive to the checklists highlighted that they made services more efficient, and were useful to identify areas requiring improvement. One service provider acknowledged that the checklists were not used to their full capacity (table 4).

##### Health promotion materials

There were positive responses to the health promotion materials from all service providers, as they believed, the materials were an ideal way to present information to parents/guardians, and indicated they had seen parents/guardians using them in the clinics. They were also used as references during the health talks (table 4).

## Discussion

This study indicated that an adaptive, clinic-led co-design process to improve local immunisation service delivery can be used to develop locally feasible tailored interventions and evaluated over a short period.

Our study showed that caregiver knowledge about vaccination can be increased, even in a relatively short period of time using simple education resources tailored to knowledge gaps, as described by our earlier assessment of barriers and enablers (to be reported separately). It was observed that overall service quality improvement was appreciated by both service providers and caregivers. While other work on routine immunisation in South Africa^28^ demonstrates the importance of minimising impact on service provider time and overload in any quality improvement intervention, our study confirmed the value of changes that can help streamline service provider activities or at least fit into existing work patterns. In other LMIC settings, communication to whole communities,^29^ plays an important role in improving immunisation uptake.

Overall, there was increased service provider awareness of the need to improve immunisation services in each clinic, which we attribute to the mix of interventions deployed. Radio sessions and immunisation education materials were deemed most useful by both service providers and caregivers. They valued the simplified presentation of educational information in both. Users valued the two-way interaction and engagement in our radio sessions, aspects that should be preserved in future iterations. Apart from the parent checklists which were unsuccessfully implemented due to limited distribution; all of the other interventions received a positive response from service users and providers. It was clear that the level of engagement service providers had with each other, and their commitment to improving immunisation services had the biggest impact on whether interventions were successful. In Myanmar, community checklists encouraged improved communication between service providers and caregivers, however, this was not replicated in our study, possibly due to implementation difficulties. In general, however, our findings suggest that locally adapted communication methods that increase community accountability without directly criticising service providers, can play a role in improving service uptake.^30 31^

Our study differs from TIP work elsewhere,^8 32^ which focuses more on reorganisation of services at the mid or national management levels, but shows the tailoring approach at work within front-line services. The TIP approach demonstrates the value of participatory problem analysis and co-design to guide responses; our experience affirms the particular value of co-design in devising locally relevant improvement solutions. Once clinics were aware of the barriers to service delivery, and engaged with the process of intervention development to address these barriers, they became more invested in providing a good quality service. Our findings also echoed those of other applications of EBCD and participatory approaches in South Africa by successfully engaging at least some service providers in ongoing quality improvement, generating greater understanding between service providers and users.^12 13^

Findings of this study highlighted improvements that seem likely to prove feasible at the clinic level elsewhere in South Africa and other LMIC settings. Overall, the co-design approach could be streamlined and used by senior immunisation service providers as a regular clinic-led quality improvement process. Development of a simplified ‘toolkit’ based on the process used in this study could assist clinics or implementation teams to improve immunisation service delivery.

One way our process could be further enhanced is with a ‘project champion’ or an ‘implementation team’ to ensure sustainability of delivery, assessment and evaluation of routine immunisation services. Another is to test application to other primary healthcare clinic services, such as HIV/TB care, to optimise UHC. Recommendations for further research to improve immunisation service delivery at the clinic level are detailed in online supplemental table 3.

Some limitations of this study largely related to the difficulty in fully implementing four different service improvement interventions in this setting over a short time frame. Staff turnover also affected both clinic services and the study process, with difficulty in maintaining service providers engagement in quality improvement consistently in all sites. This limited our ability to attribute changes in knowledge, opinions or behaviours to specific interventions.

## Conclusion

This study highlighted that parent knowledge and engagement, and service provider commitment to improvement in service quality could be improved via an adaptive, co-design clinic-led approach. Interventions that emerged from co-design in Khayelitsha focused on client communications and more convenient service arrangements. Prioritising engagement between service providers and parents/guardians can effectively identify tailored interventions, and be evaluated in a short period to achieve mutual commitment to strengthen childhood immunisation service delivery. This approach provides a framework to guide future local participatory action research for improving childhood immunisation service delivery and other child health services in South Africa and other under-resourced settings
