# Supplementary figures and images for: Using an adaptive, codesign approach to strengthen clinic-level immunisation services in Khayelitsha, Western Cape Province, South Africa
Source: BMJ Glob Health. 2021 Mar 24;6(3):e004004. doi: 10.1136/bmjgh-2020-004004 (PMC7993221; doi:10.1136/bmjgh-2020-004004)

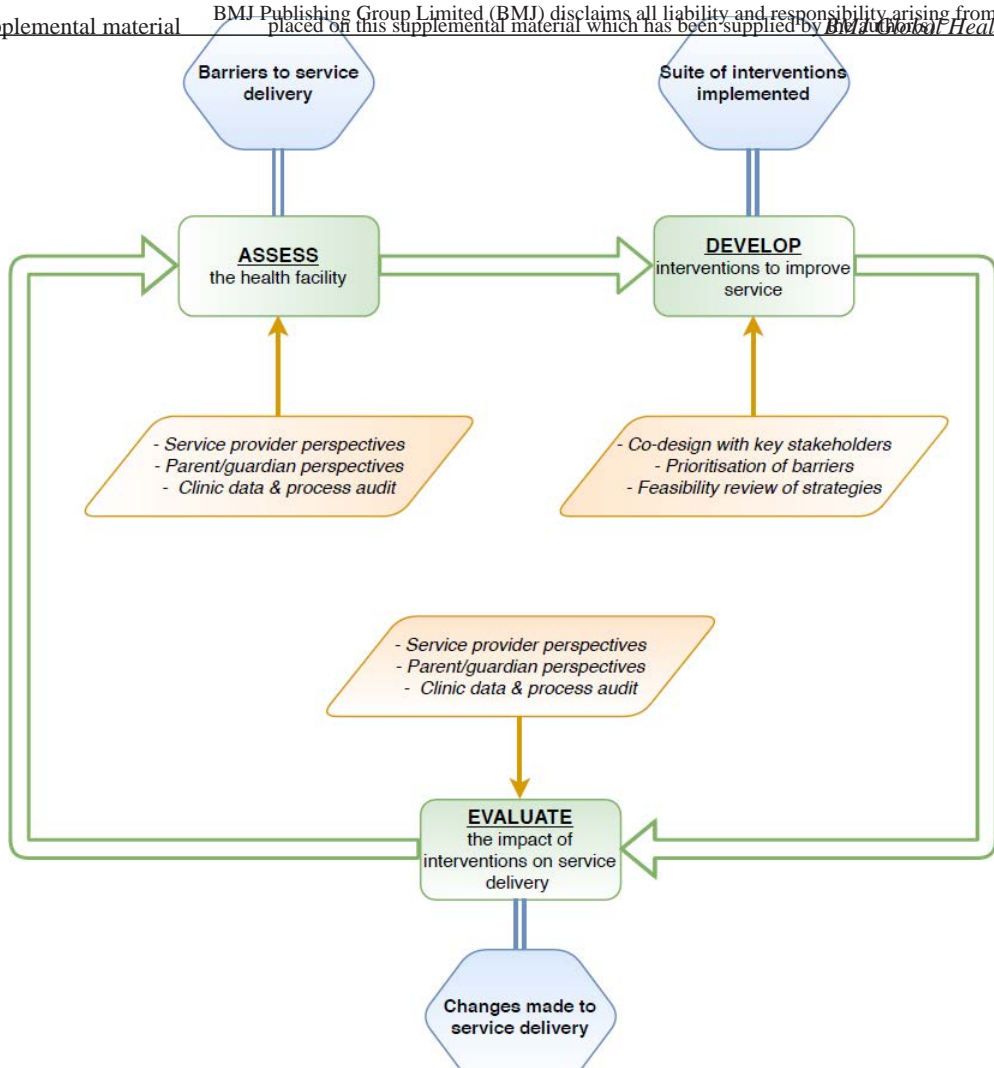

Supplement: Supplementary data [file bmjgh-2020-004004supp001.pdf]

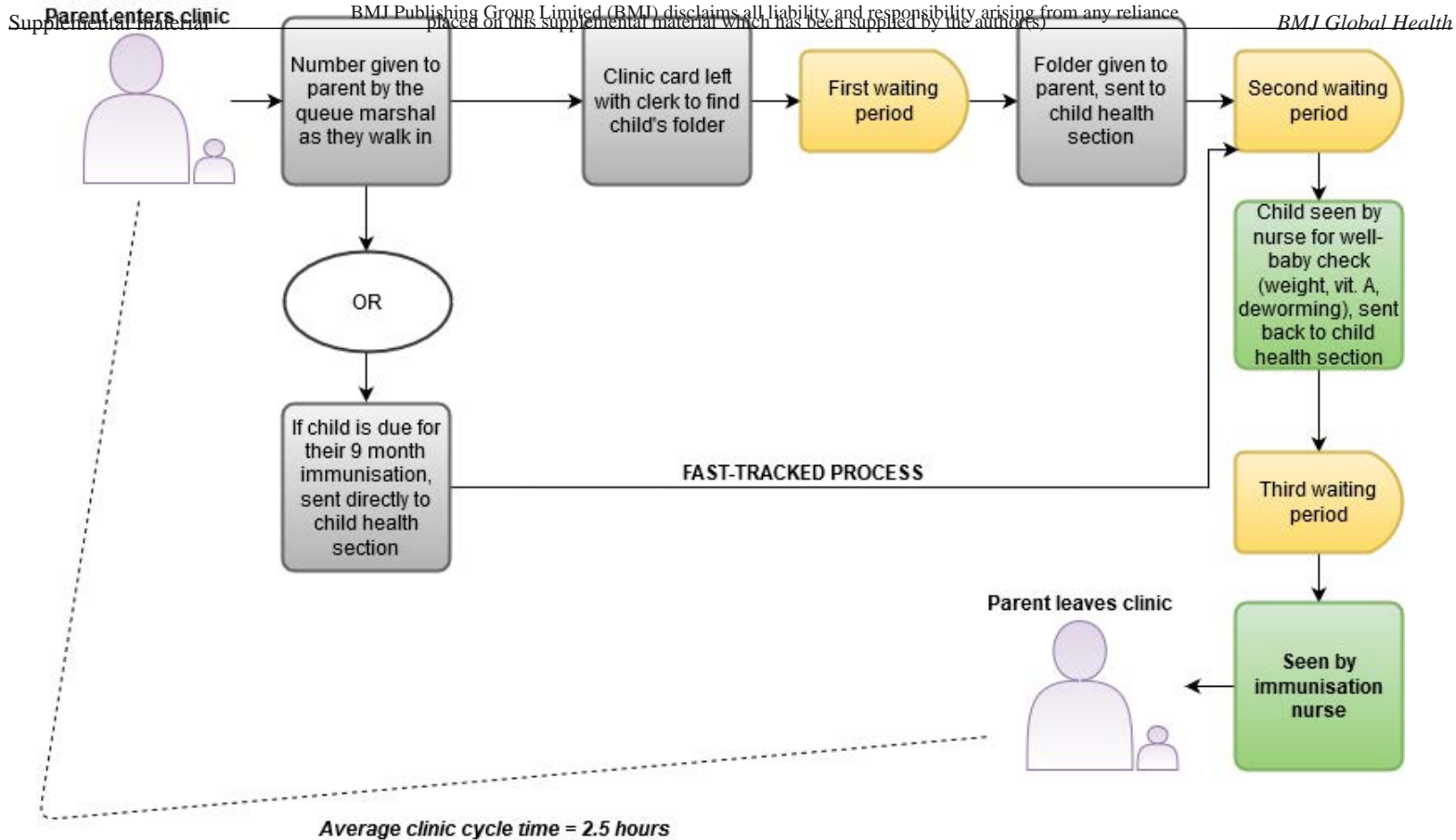

Supplement: Supplementary data [file bmjgh-2020-004004supp006.pdf]
