# Supplementary material for: Using an adaptive, codesign approach to strengthen clinic-level immunisation services in Khayelitsha, Western Cape Province, South Africa
Source: BMJ Glob Health. 2021 Mar 24;6(3):e004004. doi: 10.1136/bmjgh-2020-004004 (PMC7993221; doi:10.1136/bmjgh-2020-004004)
Supplement: Supplementary data [file bmjgh-2020-004004supp002.pdf]

Supplementary tables

Supplementary table 1. Framework for the Khusela Immunisation Study approach

<table>
<tr><th>ASSESS the health facility</th><th>DEVELOP interventions to improve service</th><th>EVALUATE the impact of strategy on service delivery</th></tr>
<tr><td>INPUT:
Clinic data assessment
Clinic process observation
Focus groups and surveys</td><td>INPUT:
Feasibility review of strategy implementation</td><td>INPUT:
Clinic data assessment
Clinic process observation
Focus groups and surveys</td></tr>
<tr><td>Components that will be assessed:</td><td>For each potential strategy:</td><td>Components that will be assessed:</td></tr>
<tr><td rowspan="2">Immunisation strategy and program
▪ Is there a clearly outlined strategy and consistent description of processes?</td><td rowspan="3">Does the facility have sufficient provincial/sub-district support to implement the strategy?</td><td>Immunisation strategy and program</td></tr>
<tr><td rowspan="2">Information systems and decision support</td></tr>
<tr><td rowspan="2">Information systems and decision support
▪ Are there suitable information systems in place for patient management?
▪ Is there adherence to evidence-based guidelines to inform decisions?</td></tr>
<tr><td rowspan="2">Does the facility have the financial resources to support the strategy?</td><td>Self-management</td></tr>
<tr><td>Self-management
▪ Promote and support self-management for clients, i.e., are there processes in place that assist and support parents/guardians in maintaining their/their child’s health, and parental knowledge?</td><td>Links with community, other health services and resources</td></tr>
<tr><td>Links with community, other health services and resources
▪ Does the facility allow for communication and cooperation with other health centres, community-based organisations and programs?</td><td rowspan="2">Does the facility have sufficient staff to support the strategy?</td><td>Feasibility of program</td></tr>
<tr><td>Feasibility of program
▪ Is program implementation feasible given existing resources and external influences?</td><td>Quality improvement of program</td></tr>
<tr><td>Quality improvement of program
▪ Are aspects of the program monitored and evaluated to improve the quality of service delivery?</td><td rowspan="2">Does the facility have the necessary additional resources to implement the strategy?</td><td>Acceptability of vaccine program</td></tr>
<tr><td>Acceptability of vaccine program
▪ Is there a high demand or level of acceptability for the immunisation program?</td><td>Effectiveness of individual strategies implemented</td></tr>
<tr><td>OUTCOMES: Barriers to service delivery</td><td>OUTCOMES: Suite of interventions implemented</td><td>OUTCOMES: Changes made to service delivery</td></tr>
</table>

Supplementary table 2. Vaccine doses administered pre- and post-intervention implementation

	Vaccines administered	Pre-intervention n (%)	Post-intervention n (%)	Total n (%)	Difference % (95% CI)	P value (Chi square test)
**Clinic A**	DTP3 (14 weeks)	49 (22)	62 (27)	112 (25)	5 (4, 5)	0.475
	MCV1 (6 months)	56 (25)	47 (20)	103 (23)	-5 (-5, -4)	0.456
	PCV3 (9 months)	67 (30)	44 (19)	112 (25)	-11 (-12, -10)	0.117
	MCV2 (12 months)	49 (22)	78 (34)	128 (28)	12 (11, 12)	0.109
	Total # vaccines administered/month	222	232	454	-	-
**Clinic B**	DTP3 (14 weeks)	92 (28)	103 (28)	195 (28)	-1 (-1, -1)	0.991
	MCV1 (6 months)	89 (27)	92 (25)	181 (26)	-3 (-3, -3)	0.781
	PCV3 (9 months)	82 (25)	82 (22)	165 (24)	-3 (-3, -3)	0.659
	MCV2 (12 months)	62 (19)	94 (25)	156 (22)	6 (6, 7)	0.366
	Total # vaccines administered/month	325	371	696	-	-
**Clinic C**	DTP3 (14 weeks)	36 (27)	37 (26)	73 (27)	-1 (-1, -1)	0.869
	MCV1 (6 months)	35 (27)	36 (26)	71 (26)	-1 (-1, -1)	0.885
	PCV3 (9 months)	35 (26)	30 (22)	65 (24)	-5 (-5, -4)	0.564
	MCV2 (12 months)	25 (19)	37 (26)	62 (23)	8 (7, 8)	0.298
	Total # vaccines administered/month	131	140	271	-	-

Supplementary table 3. Recommendations based on findings of this study

	Recommendation	Factors needed to ensure sustainability
**Within South Africa**	Ongoing assessment and evaluation of immunisation services in Khayelitsha facilitated using the approach undertaken in this study.	A ‘project champion’ from either within the clinic, or the sub-district Health Department to facilitate the process, to ensure improvement and commitment to the project and provide support to the clinics. Responsibilities would include management of funding, development and implementation of interventions, and monitoring and evaluation of immunisation service delivery.
		An ‘implementation team’ integrated into standard clinic or sub-district policy and practice to periodically assess the clinics in their designated area, develop and implement interventions, and evaluate effectivity. The current sub-district health promoter would fit under this team.
		Expansion of the approach to include other clinic services such as HIV/TB care.
**Broader use in other low-and-middle-income (LMIC) settings**	A simplified ‘toolkit’ using a scaled down approach based on this study that would assist clinics or implementation teams to improve immunisation service delivery or other preventative, chronic, or mental health services in other regions in South Africa or other LMIC settings, similar to the urban immunisation toolkit developed by UNICEF.	Primary assessment tools including a short survey for service users and providers to determine the barriers and facilitators to immunisation service delivery, supplemented by a focus group with service users and providers if needed.
		A short period of observation of clinic activity would occur simultaneously to triangulate the approach.
		The data generated used to design interventions appropriate to the setting with key stakeholders, and implemented quickly.
		Scaled down working groups consisting of the implementation team and a representative of the targeted clinic, and interventions developed that are appropriate to the scope and budget of the clinic that do not require external funding in order to ensure the process is sustainable and feasible for the clinics.
		A short, targeted evaluation based on the initial assessment and specific to implemented interventions.
		A periodic assessment/evaluation cycle would result in barriers being easier to identify, and eliminate the need for an intensive intervention development process.
