# Supplementary material for: Using an adaptive, codesign approach to strengthen clinic-level immunisation services in Khayelitsha, Western Cape Province, South Africa
Source: BMJ Glob Health. 2021 Mar 24;6(3):e004004. doi: 10.1136/bmjgh-2020-004004 (PMC7993221; doi:10.1136/bmjgh-2020-004004)
Supplement: Supplementary data [file bmjgh-2020-004004supp003.pdf]

Nurse Immunisation Checklist

Before the immunisation session

No.	Item	Tick in the box if complete
1.	Prior notice to parents about date and time of immunisation session	☐
2.	Health Education/materials distributed before commencing the immunisation session	☐

During the immunisation session

No.	Item	Tick in the box if complete
3.	Greet the client and caregiver and ask them if they have any concerns. If necessary, check the general health status of the child.	☐
4.	Review the client's immunisation card and confirm contact details with the caregiver	☐
5.	Determine all eligible vaccinations based on the national schedule, client's age and possible contraindications	☐
6.	Record all vaccinations in register, due list, and immunisation card	☐
7.	Communicate key messages, including potential AEFIs and give parents health promotion materials if appropriate	☐
8.	Inform caregiver when the child's next immunisation is due	☐
9.	Refer parents to other community health services such as social services, the nutrition unit, or non-profit organisations if appropriate	☐

After the immunisation session

No.	Item	Tick in the box if complete
10.	List the names of children who missed vaccination and require follow up	☐
11.	Take appropriate action and prepare list of children to be vaccinated next month, including children that missed their vaccination this month	☐

Clerk Immunisation Checklist

Before daily immunisation sessions

No.	Item	Tick in the box if complete
1.	Prior notice to parents about date and time of immunisation session	☐

During daily immunisation sessions

No.	Item	Tick in the box if complete
2.	Greet the client and caregiver and ask them if they have an immunisation appointment	☐
3.	Ensure contact details of caregiver are up to date	☐
4.	Remind client to make an appointment for the next immunisation session to save time and skip the queue	☐
5.	Record all vaccinations in PREHMIS	☐
6.	Inform caregiver when the child's next immunisation is due	☐

After daily immunisation sessions

No.	Item	Tick in the box if complete
7.	List the names of children who missed vaccination and require follow up	☐
8.	Take appropriate action and prepare list of children to be vaccinated next month, including children that missed their vaccination this month	☐
9.	Prepare folders for the next day's immunisation appointments	☐
