# Supplementary material for: Using an adaptive, codesign approach to strengthen clinic-level immunisation services in Khayelitsha, Western Cape Province, South Africa
Source: BMJ Glob Health. 2021 Mar 24;6(3):e004004. doi: 10.1136/bmjgh-2020-004004 (PMC7993221; doi:10.1136/bmjgh-2020-004004)
Supplement: Supplementary data [file bmjgh-2020-004004supp004.pdf]

BMJ Publishing Group Limited (BMJ) disclaims all liability and responsibili
placed on this supplemental material which has [illegible]

Immunisation Program Feedback

The purpose of this form is to help us understand what your experience with the immunisation program at our clinic was like. Please answer the questions below to help us decide how we can provide you with a better immunisation service.

No.	Item	Circle one box Yes/Unsure/No		
1.	Did you feel the immunisation provider treated you respectfully?	✓	?	×
2.	Did you understand which vaccinations your child was given (that is: against what diseases)?	✓	?	×
3.	Was the vaccination/s your child received recorded on your vaccination card/Road to Health booklet?	✓	?	×
4.	Do you understand when your child should come back for the next vaccination?	✓	?	×
5.	Do you understand the possible side-effects of the vaccination received?	✓	?	×
6.	Are you aware of other community health services such as social services, the nutrition unit, or non-profit organisations that you may be eligible for?	✓	?	×
7.	Have you ever attended a health education talk about immunisation in the clinic?	✓	?	×
8.	Have you ever received/seen any posters or pamphlets about immunisation in the clinic?	✓	?	×
9.	Have you ever heard anything about immunisation on your local radio station?	✓	?	×
10.	Did you feel the service was delivered appropriately and effectively?	✓	?	×
11.	Did you feel that the clinic provided a good quality service?	✓	?	×

Additional feedback: ______________________________

____

____

____

____

____

Thank you for your help ☺
