# Supplementary material for: Using an adaptive, codesign approach to strengthen clinic-level immunisation services in Khayelitsha, Western Cape Province, South Africa
Source: BMJ Glob Health. 2021 Mar 24;6(3):e004004. doi: 10.1136/bmjgh-2020-004004 (PMC7993221; doi:10.1136/bmjgh-2020-004004)
Supplement: Supplementary data [file bmjgh-2020-004004supp005.pdf]

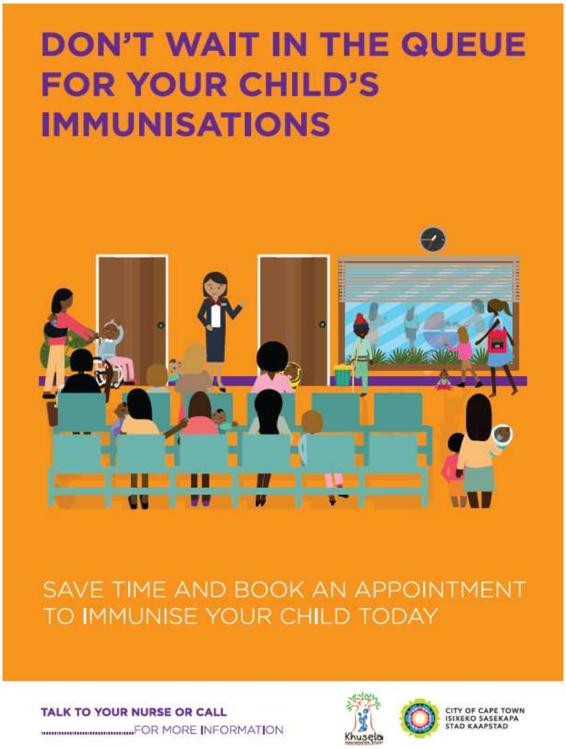
DON'T WAIT IN THE QUEUE FOR YOUR CHILD'S IMMUNISATIONS
SAVE TIME AND BOOK AN APPOINTMENT TO IMMUNISE YOUR CHILD TODAY
TALK TO YOUR NURSE OR CALL
..............................FOR MORE INFORMATION
Khusela
CITY OF CAPE TOWN
ISIXEKO SASEKAPA
STAD KAAPSTAD

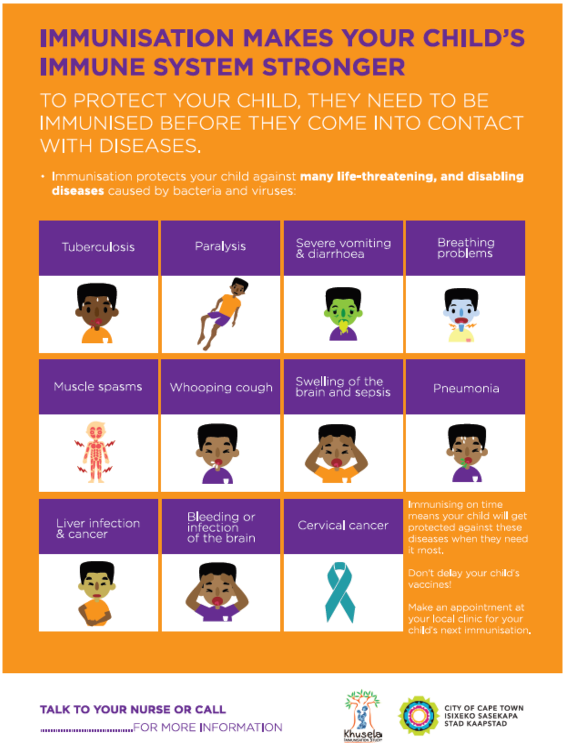
IMMUNISATION MAKES YOUR CHILD'S IMMUNE SYSTEM STRONGER
TO PROTECT YOUR CHILD, THEY NEED TO BE IMMUNISED BEFORE THEY COME INTO CONTACT WITH DISEASES.
• Immunisation protects your child against many life-threatening, and disabling diseases caused by bacteria and viruses:
Tuberculosis
Paralysis
Severe vomiting & diarrhoea
Breathing problems
Muscle spasms
Whooping cough
Swelling of the brain and sepsis
Pneumonia
Liver infection & cancer
Bleeding or infection of the brain
Cervical cancer
Immunising on time means your child will get protected against these diseases when they need it most.
Don't delay your child's vaccines!
Make an appointment at your local clinic for your child's next immunisation.
TALK TO YOUR NURSE OR CALL
..............................FOR MORE INFORMATION
Khusela
CITY OF CAPE TOWN
ISIXEKO SASEKAPA
STAD KAAPSTAD

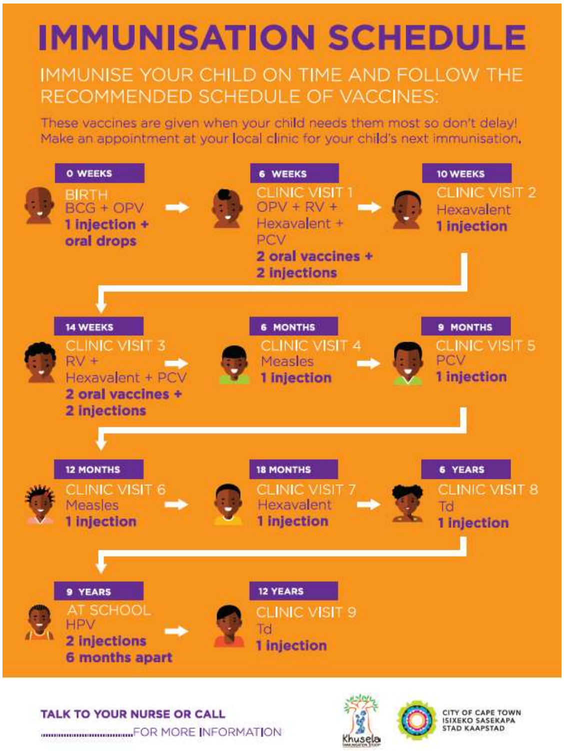
IMMUNISATION SCHEDULE
IMMUNISE YOUR CHILD ON TIME AND FOLLOW THE RECOMMENDED SCHEDULE OF VACCINES:
These vaccines are given when your child needs them most so don't delay! Make an appointment at your local clinic for your child's next immunisation.
0 WEEKS
BIRTH
BCG + OPV
1 injection + oral drops
6 WEEKS
CLINIC VISIT 1
OPV + RV + Hexavalent + PCV
2 oral vaccines + 2 injections
10 WEEKS
CLINIC VISIT 2
Hexavalent
1 injection
14 WEEKS
CLINIC VISIT 3
RV + Hexavalent + PCV
2 oral vaccines + 2 injections
6 MONTHS
CLINIC VISIT 4
Measles
1 injection
9 MONTHS
CLINIC VISIT 5
PCV
1 injection
12 MONTHS
CLINIC VISIT 6
Measles
1 injection
18 MONTHS
CLINIC VISIT 7
Hexavalent
1 injection
6 YEARS
CLINIC VISIT 8
Td
1 injection
9 YEARS
AT SCHOOL
HPV
2 injections
6 months apart
12 YEARS
CLINIC VISIT 9
Td
1 injection
TALK TO YOUR NURSE OR CALL
..............................FOR MORE INFORMATION
Khusela
CITY OF CAPE TOWN
ISIXEKO SASEKAPA
STAD KAAPSTAD

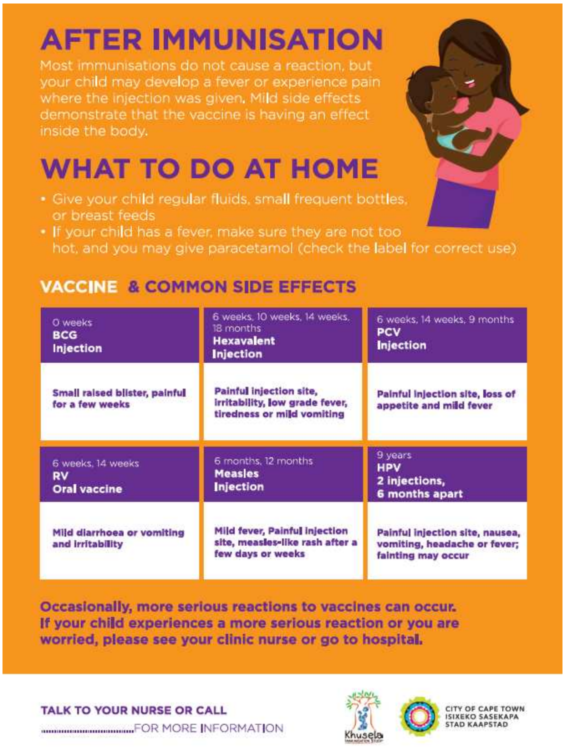
AFTER IMMUNISATION
Most immunisations do not cause a reaction, but your child may develop a fever or experience pain where the injection was given. Mild side effects demonstrate that the vaccine is having an effect inside the body.
WHAT TO DO AT HOME
• Give your child regular fluids, small frequent bottles, or breast feeds
• If your child has a fever, make sure they are not too hot, and you may give paracetamol (check the label for correct use)
VACCINE & COMMON SIDE EFFECTS
0 weeks BCG Injection
Small raised blister, painful for a few weeks
6 weeks, 10 weeks, 14 weeks, 18 months Hexavalent Injection
Painful injection site, irritability, low grade fever, tiredness or mild vomiting
6 weeks, 14 weeks, 9 months PCV Injection
Painful injection site, loss of appetite and mild fever
6 weeks, 14 weeks RV Oral vaccine
Mild diarrhoea or vomiting and irritability
6 months, 12 months Measles Injection
Mild fever, Painful injection site, measles-like rash after a few days or weeks
9 years HPV 2 injections, 6 months apart
Painful injection site, nausea, vomiting, headache or fever; fainting may occur
Occasionally, more serious reactions to vaccines can occur. If your child experiences a more serious reaction or you are worried, please see your clinic nurse or go to hospital.
TALK TO YOUR NURSE OR CALL
..............................FOR MORE INFORMATION
Khusela
CITY OF CAPE TOWN
ISIXEKO SASEKAPA
STAD KAAPSTAD
